# *Allium macrostemon* Saponin Inhibits Activation of Platelet via the CD40 Signaling Pathway

**DOI:** 10.3389/fphar.2020.570603

**Published:** 2021-01-13

**Authors:** Sisi Ling, Lijun Jin, Shizheng Li, Fangcheng Zhang, Qiong Xu, Mingke Liu, Xuke Chen, Xiaolin Liu, Jielei Gu, Shiming Liu, Ningning Liu, Wenchao Ou

**Affiliations:** Department of Cardiology, Guangdong Key Laboratory of Vascular Diseases, State Key Laboratory of Respiratory Disease, Guangzhou Institute of Cardiovascular Disease, The Second Affiliated Hospital of Guangzhou Medical University, Guangzhou, China

**Keywords:** *Allium macrostemon* saponin, platelet activation, CD40, TRAF2, degradation

## Abstract

*Allium macrostemon* saponin is a traditional Chinese medicine that exhibits anti-atherosclerosis effects. However, the mechanism of its action has not been fully clarified. Platelet activation induced by CD40L plays an important role in the process of atherosis. In the present study, we demonstrate for the first time that *A. macrostemon* saponin inhibits platelet activation induced by CD40L. Moreover, the effects of saponin on platelet activation were achieved by activation of the classical CD40L-associated pathway, including the PI3K/Akt, MAPK and NF-κB proteins. In addition, the present study further demonstrated that saponin exhibited an effect on the TRAF2-mediated ubiquitination degradation, which contributed to the inhibition of the CD40 pathway and its downstream members. The findings determine that *A. macrostemon* saponin inhibits activation of platelets via activation of downstream proteins of the CD40 pathway. This in turn affected TRAF2-associated ubiquitination degradation and caused an anti-thrombotic effect.

## Introduction

Atherosclerotic cardiovascular disease is the leading cause of death and morbidity worldwide and more than 80% of CVD mortality cases occur in developing countries according to a previous study (Steg et al., 2011). Atherosclerosis is the pathological basis of CVD and is considered a chronic inflammatory disease caused by complex immune and metabolic processes, in which platelet activation plays an important role. Platelet activation not only stimulates autoinflammatory releasing inflammatory mediators but also releases active molecules, such as thrombins to promote the progression of atherosclerosis (Ahmadsei et al., 2015; Ziegler et al., 2019). The CD40L/CD40 pathway has been shown to have important effects on platelet activation leading to increase of thrombus formation in response to vascular injury (Aukrust et al., 2004).

CD40 is a member of the tumor necrosis factor receptor (TNF-R) super family and binds to the CD40L, a 39KD transmembrane glycoprotein, which is biologically active (Lievens et al., 2009). During the process of platelet activation, the CD40L/CD40 pathway induces inflammation directly resulting in abundant expression of adhesion molecules (selectin,VCAM-1,ICAM-1) and stimulates production of chemokines as well as a wide range of cytokines (Aukrust et al., 2004; Lievens et al., 2009). Binding the CD40L to CD40 induces the classical CD40-related signaling pathway. Initially, sCD40L enhances agonist-induced activation and aggregation of human platelets via the CD40-mediated tumor necrosis factor receptor–associated factor (TRAF)-2/Rac1/p38 mitogen-activated protein kinase (MAPK)-dependent pathway, which results in platelet activation (Yacoub et al., 2010). In addition, accumulating evidence has suggested that the PI3K/Akt pathway has also emerged as a crucial player in platelet activation, which contributes to regulate integrin function and participates in platelet activation as well as aggregation (Martin et al., 2010; Guidetti et al., 2015; Gupta et al., 2015; Kuijpers et al., 2015; Wan et al., 2018). In addition, the CD40L/CD40 complex induces activation of nuclear factor kappa B (NF-κB), which involves both IKK and IKB in the NF-κB signaling pathway. The latter exhibits a different non-genomic function in macrostemon platelets and induces inflammation and thrombosis (Hachem et al., 2012; Karim et al., 2015; Paul et al., 2017; Xu et al., 2017; Kojok et al., 2019). In addition, the conjugation of TRAF protein (E3 ligase) with the CD40 receptor plays an important role in the CD40L/CD40 pathway, by mediating the activation of the canonical and non-canical NF-κB pathways as well as the MAPK pathway in response to CD40 (Hadweh et al., 2014; de Jong et al., 2016; El Hokayem et al., 2017; Chen et al., 2018; Seijkens et al., 2018; Johansson et al., 2019; Unsworth et al., 2019).

*Allium macrostemon* is a dry bulb of Allium macrostemon Bge, which belongs to the lily family and is used in the clinical treatment of CVD. However, its pharmacological mechanism has not been clarified. Our research group has studied extensively the anti-atherosclerotic mechanism of *A. macrostemon* saponin and the extract of *A. macrostemon*. In our preliminary study, it was found that *A. macrostemon* saponin not only inhibited ADP-induced platelet activation and aggregation, but also prevented the release of CD40L, which played an anti-antherosclerotic role (Ou et al., 2012; Ou et al., 2016) These preliminarily studies clarified the inhibition of ADP-induced platelet activation by saponins (Ou et al., 2012). Based on the pharmacological characteristics of the multitarget of the *A. macrostemon* saponins, we further hypothesized that saponins may also influence platelet activation through the CD40L/CD40 pathway. In the present study, we verified that *A. macrostemon* saponin inhibited platelet activation via the CD40-associated signaling pathway.

## Materials and Methods

### Chemicals and Antibodies

Macrostemon is the dry bulb of *A. macrostemon* Bunge (Ou et al., 2012; Ou et al., 2016; Feng et al., 2019). *A. macrostemon* saponin was provided from professor Haifeng Chen (XiaMen University) (Ou et al., 2012) and Macrostemon was purchased from Bencaopu online store (ECN000168). In addition, *A. macrostemon* saponin whose purity exceeded 90% was separated and identified by professor Qishi Sun (College of traditional Chinese medicine, Shenyang pharmaceutical University) using an HPLC-MS method.

The study protocol was approved by the second affiliated hospital of the Guangzhou medical University, at Guangzhou, China. The donors were asked to sign a written informed consent for their participation in the study.

Table liquid was purchased from Procell (China - Wuhan), whereas the anti-VCAM -1 and the anti-ICAM-1 antibodies were provided from Abcam (United Kingdom). Anti-p-JNK, anti-p-NF-κB, anti-CD40L, anti-β-actin, anti-p-p38 and anti-IκBα antibodies were from Cell Signaling Technology, (United States). Vcam-1 and ICAM-1 ELISA kits were from Dakota Biotechnology Co., Ltd. (China - Beijing).

### Platelet Isolation and Extraction

The present study selected 30 donors based on the following eligibility criteria: 1) Age: 20–65 years old. 2). Disease history: No history of coronary heart disease, diabetes, stroke or internal bleeding within six months, no serious infection, absence of liver disease, kidney disease, tumor and other diseases. 3) History of drug use: The donors did not receive anti-platelet, anti-coagulation and anti-infection drugs and/or other drugs for nearly three months. The present study used BD citrate sodium anticoagulant blood vessels (1:9) for extraction of peripheral blood (4 ml). Total blood was extracted following centrifugation at room temperature (1,000 g) for 10 min and subsequently the top layer of the stratified blood samples, called platelet-rich plasma, was collected gently. Subsequently, PRP was centrifuged at room temperature (1,000 g for 5 min) in order to obtain the platelet pellet. The latter was washed with Tyrode’s albumin buffer and centrifuged at 1,000 g for 5 min to obtain washed platelets suspended in Tyrode’s albumin buffer.

### Pretreatment of Platelet

The following groups were used in our study: 1) control: The washed platelet without any treatment was used as the control group. 2) CD40L + *A. macrostemon* saponin (200 μg/ml): Washed platelets pretreated with saponin (200 μg/ml) for 1 h was activated by CD40L (50 μM) for 30 min. 3) CD40L + *A. macrostemon* saponin (200 μg/ml): Washed platelets pretreated with saponin (400 μg/ml) for 1 h was activated by CD40L (50 μM) for 30 min. 4) MG132: Washed platelets which were preprocessed with MG132 (10 μM) for 1 h pretreated with saponin (400 μg/ml) for 1 h and subsequently activated by CD40L (50 μM) for 30 min.

### Western Blotting

Each group of washed platelets was removed by the lysate to obtain the platelet protein. The protein levels were determined by western blotting as we previously reported (Liao et al., 2019; Zhang et al., 2020), following quantification of the protein concentration with the BCA method. Initially, The sodium dodecyl sulfate (SDS)-polyacrylamide gels with different concentrations were prepared according to the molecular weight of the target protein. Subsequently, the platelet proteins were separated by gel electrophoresis and transferred electrophoretically on nitrocellulose membrans. The membrans were blocked with 5% skimmed milk and subsequently incubated with primary antibodies in blocking buffer for one night. Finally, the blots were incubated with horseradish peroxidase (HRP)-conjugated secondary antibody and developed by the ECL method. Moreover, The gray value of each strip was analyzed with the Image J software and the protein expression levels were determined by the ratio of the expression of the target protein to that of the internal reference protein β-actin.

### Detection of TXB2 by the ELISA Assay

Each group of washed platelet was centrifuged at 1,000 g for 10 min in order to obtain the supernatant. The levels of the TXB2 protein in the supernatant were detected by referring to the instructions of the ELISA assay kit as we previously reported (Liu et al., 2017; Xu et al., 2019). The absorbance was detected at 450 nm. Finally, the concentration levels of TXB2 were calculated according to the standard curve.

### Flow Cytometry

CD62p-APC was used to monitor platelet activation. A total of 10 μl sample was obtained from different treatment groups of washed platelets and was incubated in the dark for 30 min at room temperature and subsequently fixed with 1% paraformaldehyde. The samples were analyzed by flow cytometry as we previously reported (Hu et al., 2018).

### Detection of NF-κB by the NF-κB Activity Kit

The preparation of samples was performed as follows: the samples were performed through the total cell extraction method. Initially peripheral venous blood (4 ml) was collected and centrifuged at 1,000 g for 10 min at room temperature to obtain platelet-rich plasma (PRP). Subsequently, the platelets were separated from PRP following centrifugation at 1,000 g for 5 min at room temperature. The washed platelets were obtained by the Tyrode solution. The washed platelets were treated with saponin for 1 h as well as with CD40L for 30 min. Finally, in order to obtain the whole cell extract, the washed platelets were incubated at 4°C for 1 h and centrifuged at 12,000 g for 15 min. Each group of samples was used to detect the NF-κB activity by the NF-κB transcription factor assay kit according to the manufacturer’s instructions.

### Statistical Analyses

Experimental primary data analyzed by professional The SPSS 16.0 software were used for experimental data analysis. Data were presented as mean ± standard deviation (SD). Differences between the groups were determined by one-way analysis of variance (ANOVA) and t-test. *p* value of <0.05 was considered to be statistically significant.

## Results

### Saponin Inhibits the Activation of Platelets by the CD40-Associated Pathway

**FIGURE 1:**
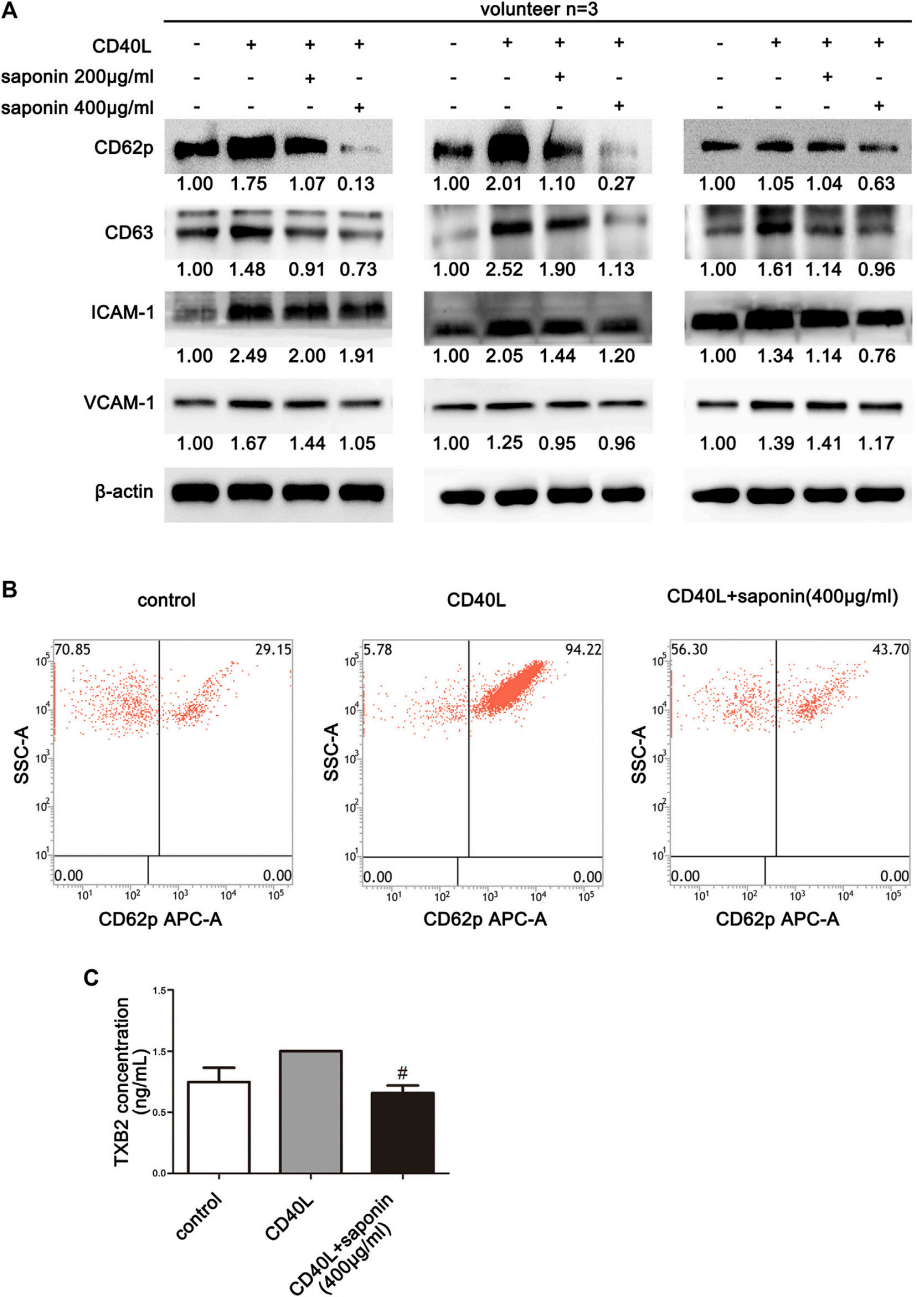
Inhibition of platelet activation by saponin. The data from three volunteers are presented. **(A)** Western blotting demonstrating the expression levels of the inflammatory mediators, such as ICAM-1, VCAM-1 and/or the platelet activation marker, such as CD62p and CD63, and the protein gray value of each group was compared to the control. Washed platelets were pretreated with saponin (200 μg/ml, 400 μg/ml) or not for 1 h, then stimulated with sCD40L (10 μM) for 30 min, platelets lysates were resolved in 10% SDS-PAGE or 12% SDS-PAGE and assessed for target protein. **(B)** The results indicated the measurements of CD62p on the surface of washed platelets by flow cytometry. **(C)** ELISA assay detected washed platelets supernatant to indicate release levels of TXB2. CD40L was used as a positive control for TXB_2_ release by platelets. Following saponin treatment (400 μg/ml) TXB2 expression was reduced compared with that noted following CD40L treatment (10 μM). #*p* < 0.05 vs. CD40L.

To explore the potential inhibition of platelet activation by CD40L, the ability of saponin to suppress platelet aggregation and the effects on activated platelet induction by CD40L were examined in a preliminary study. Initially, the platelets were pretreated with saponin and allowed to interact with CD40L. In addition, platelets without treatment were used as the control group. The results from three volunteers were examined and the results indicated that the platelet activity markers CD62P and CD63 were inhibited by saponin treatment, following activation of the platelets by CD40L resulting in the upregulation of platelet active marker expression. These preliminary findings suggested that Saponin may possibly inhibit the activation of platelets (Figures 1A,B). In addition, CD40L, which has been reported to induce the inflammatory responses of platelets, was confirmed to stimulate the production of inflammatory mediators, such as ICAM-1 and VCAM-1 in platelets. In contrast to these observations, the expression levels of both inflammatory mediators were downregulated in active platelets pretreated by saponin (Figures 1A,B). Therefore, the current study indicated that saponin could influence platelet activation to modulate the induction of inflammation. Moreover, in order to further verify the inhibition of platelet activation by saponin, we examined whether saponin could affect the release of TXB2 and platelet-derived microvesicles (PMVs). It is well known that active platelets can release large amounts of TXB2 and PMVs. The ELISA assay was conducted to confirm that saponin reduced the release of TXB2 and PMVs (Figure 1C and Supplementary Figure S1). We also found that the platelet activity markers CD63 and TXB2 which induced by ADP were inhibited by saponin treatment (Supplementary Figure S2). The results demonstrated that saponin inhibited the activation of platelets.

### Saponin Reduced the Activation of the PI3K/Akt Pathway

**FIGURE 2:**
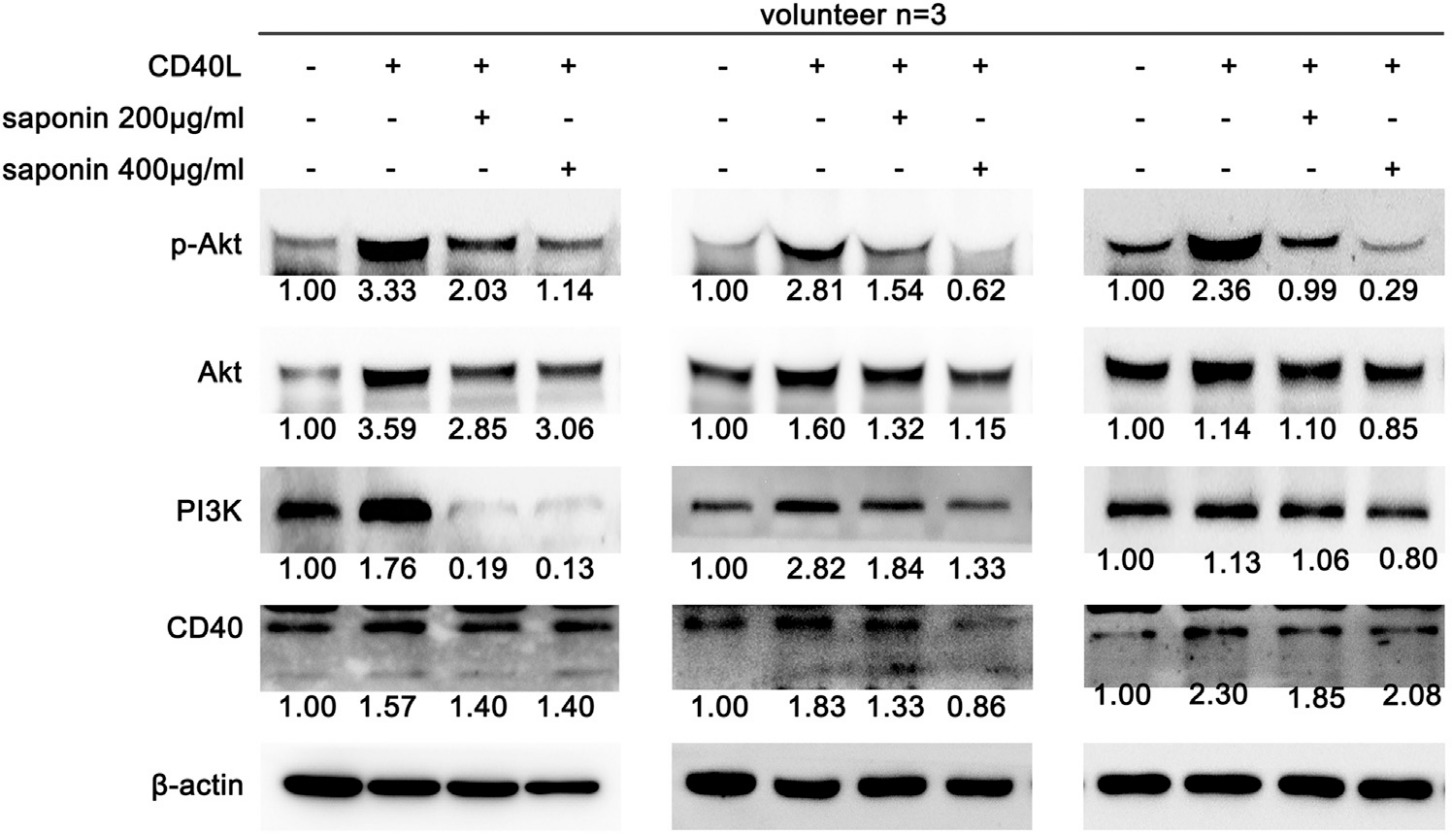
Inhibition of the constitutive expression of the PI3K/Akt pathway by saponin causes decreased platelet activity. The results of three volunteers are shown. Western blotting indicated protein expression levels of CD40, as well as of the PI3K/Akt signaling molecules PI3K, Akt and P-Akt, and the protein gray value of each group was compared to the control. Washed platelets were pretreated with saponin (200 μg/ml, 400 μg/ml) or not for 1 h, then stimulated with sCD40L (10 μM) for 30 min, platelets lysates were resolved in 12% SDS-PAGE and assessed for target protein.

Since saponin inhibited the activation of platelets, its associated mechanism of action and its effects on the CD40-associated pathway were explored. The data indicated that saponin affected the classical CD40-associated pathway, such as the PI3K/Akt, the MAPK and the NF-κB pathways. Initially, the potential role of saponin in the PI3K/Akt pathway was investigated and the data indicated that this compound could downregulate the expression levels of CD40, PI3K and p-Akt (Figure 2). The results suggested that saponin reduced the activation of the PI3K/Akt pathway.

### Saponin Reduced the Activation of the MAPK Pathway

**FIGURE 3:**
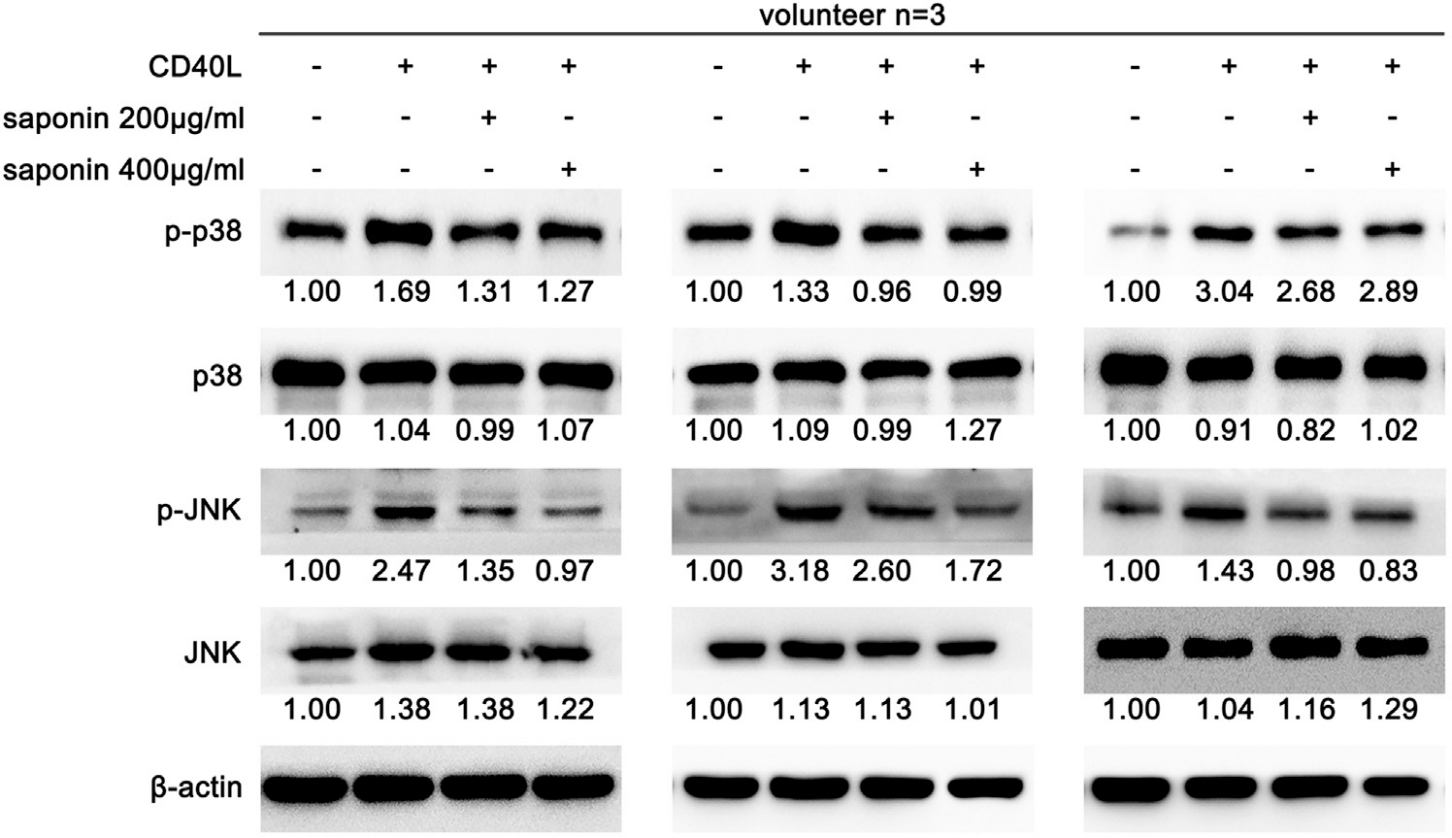
Saponin reduces the protein expression levels of the MAPK pathway in platelets. The results of three volunteers are shown. Western blotting indicated protein expression levels of CD40, as well as of the p-p38 and p-JNK, and the protein gray value of each group was compared to the control. Washed platelets were pretreated with saponin (200 μg/ml, 400 μg/ml) or not for 1 h, then stimulated with sCD40L (10 μM) for 30 min, platelets lysates were resolved in 12% SDS-PAGE and assessed for target protein.

The current study further determined the effects of saponin on the MAPK pathway. Saponin further triggered the activation of the MAPK pathway by CD40. It was shown that the expression levels of the MAPK pathway proteins p-p38 and p-JNK were increased following treatment with CD40L. However, all of these proteins were decreased in active platelets treated with saponin, as demonstrated by western blotting (Figure 3). These results supported the hypothesis that saponin reduced the activation of the MAPK pathway.

### Saponin Reduced the Activation of the NF-κB Pathway

**FIGURE 4:**
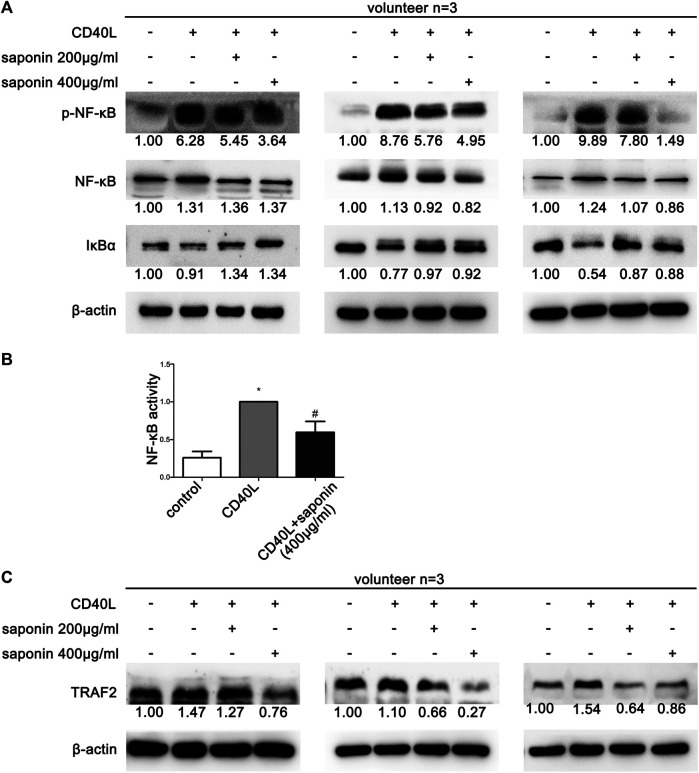
Saponin downregulated the protein expression levels of the NF-κB pathway proteins and caused the inhibition of the CD40 signaling pathway via induction of TRAFs. Saponin downregulated the protein expression levels of the NF-κB pathway proteins and caused the inhibition of the CD40 signaling pathway via induction of TRAFs. The results from three volunteers are shown. **(A,C)** Western blotting indicated protein expression levels of the NF-κB signaling molecules NF-κB, p-NF-κB and IκB, as well as the E3 ligase TRAF 2, and the protein gray value of each group was compared to the control. Washed platelets were pretreated with saponin (200 μg/m, 400 μg/ml) or not for 1 h, then stimulated with sCD40L (10 μM) for 30 min, platelets lysates were resolved in 12% SDS-PAGE and assessed for target protein. **(B)** Platelets lysates were detected by NF-κB activity assay and bars represented different NF-κB activity. *p < 0.05 vs. control. #*p* < 0.05 vs. CD40L.

Previous data demonstrated that saponin could extensively inhibit the CD40-associated signaling pathways. Therefore, we explored the effects of saponin on the NF-κB pathway using western blotting. In line with our expectations, the protein expression levels of p-NF-κB were upregulated following treatment by CD40L. Concomitantly, the protein expression levels of IκB-α were downregulated under the same treatment conditions. In contrast to these findings, the reverse results were obtained with regard to the NF-κB pathway in platelets pretreated with saponin (Figure 4A). The NF-κB activity test results further suggested that saponin reduced NF-κB activity (Figure 4B). In conclusion, saponin inhibited the activation of platelets via the CD40-associated classical signaling pathway, which included the PI3K/Akt, the MAPK and the NF-κB pathway. In addition, TRAF2 is a E3 ligase conjugated to CD40, which exhibited decreased levels in platelets pretreated with saponin. However, its protein expression levels were increased following treatment by CD40L in our results. It was shown that saponin may regulate the aforementioned CD40-related signaling pathway via TRAF2 (Figure 4C).

### Saponin Regulates TRAF2 Stability by Affecting its Ubiquitination and Degradation

**FIGURE 5:**
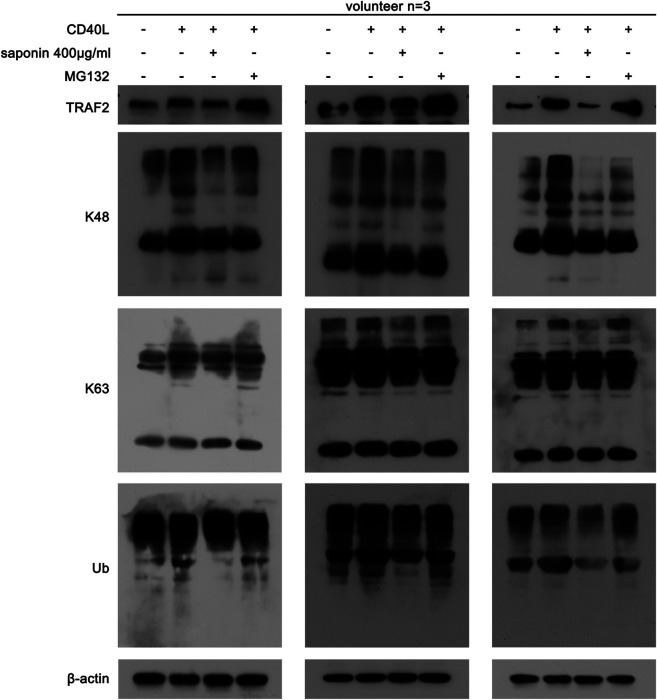
Saponin-mediated inhibition of ubiquitination is associated with the Ubiquitin E3 ligase TRAF2. Washed platelets were pretreated with saponin (400 μg/ml) or not for 1 h, besides washed platelets were treated with MG132 (10 μM) for 30 min after pretreated with saponin (400 μg/ml) for 1 h in MG132 group, then stimulated with sCD40L (10 μM) for 30 min, platelets lysates were resolved in 12% SDS-PAGE and assessed for protein level of TRAF2, K48, K63 and Ub.

Following the initial findings of the effects of saponin on TRAF2, we further studied whether this compound could regulate the CD40-associated signaling pathway by affecting TRAF2-associated ubiquitination. Saponin may affect the poly ubiquitination modification of platelets, which resulted in changing the acting site of K63 and of K48. As shown in Figure 5, saponin reduced the protein expression levels of K63 and K48, which were increased in the active platelet group following treatment by CD40L, while MG132 could reverse the effects of saponin on the degradation of these proteins. Moreover, the results demonstrated that saponin exhibited the same effect on ubiquitin as K63 and K48. Based on these results, it is reasonable to speculate that saponin could regulate the CD40-associated signaling pathway by affecting the poly ubiquitination modification of TRAF2, thereby inhibiting the activation of platelets.

### Proteasome Inhibition can Reverse Platelet Activity

**FIGURE 6:**
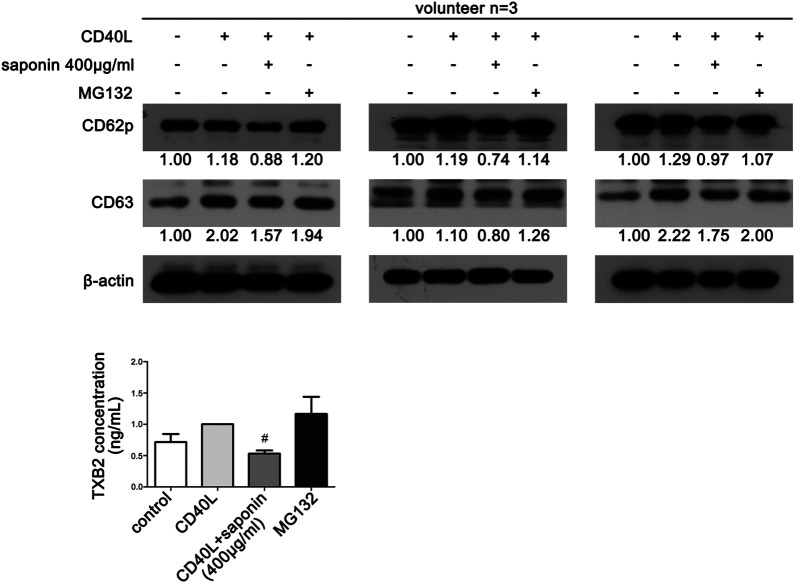
MG132 reverses saponin-mediated inhibition of platelet activation. **(A)** Western blotting indicated the protein expression of platelet activation markers CD62P and CD63. Washed platelets were pretreated with saponin (400 μg/ml) or not for 1 h, besides washed platelets were treated with MG132 (10 μM) for 30 min after pretreated with saponin (400 μg/ml) for 1 h in MG132 group, then stimulated with sCD40L (10 μM) for 30 min, platelets lysates were resolved in 12% SDS-PAGE. **(B)** TXB2 was released when platelet was activated by CD40L. ELISA assay detected washed platelets supernatant to indicate differrent release levels of TXB2 following different treatment of CD40L, saponin (400 μg/ml) and MG132 (10 μM). #*p* < 0.05 vs. CD40L.

Finally, in order to verify the effects of saponin on platelet activation and ubiquitination, we investigated whether MG132 could reverse platelet activity. Western blot analysis demonstrated that the protein expression levels of both CD63 and CD62p were upregulated following pretreatment with MG132. Both of them were downregulated following treatment by saponin (Figure 6A). Similarly, MG132 increased the release of TXB2, which was blocked by saponin (Figure 6B).

## Discussion

Platelet activation has an important effect on promoting atherosclerosis (Steg et al., 2011; Ahmadsei et al., 2015). Therefore, the reduction in the activation of platelets is the focus of the present study. *A. macrostemon* saponin exhibits multiple biological effects (Chen et al., 2007; Xie et al., 2008; Chen et al., 2009; Ou et al., 2012; Feng et al., 2019). In the present study, we studied the inhibition of sCD40L-induced platelet activation by saponin, while it was determined that saponin reduced platelet activation in our previous study (Ou et al., 2012). In this study, the experimental results primarily indicated that sCD40L induced the activation and inflammation of platelets, which caused an upregulation in the expression levels of platelet activation markers, such as CD62P, CD63 and of the inflammatory mediator, such as VCAM-1 and ICAM-1. In addition, sCD40L promoted the release of TXB2 in accelerating thrombosis of plaque. However, it was found that *A. macrostemon* saponin could inhibit sCD40L-induced platelet activation suggesting a potential anti-atherosclerotic mechanism. This conclusion suggested that sCD40L-induced platelet activation may be a novel anti-atherosclerotic mechanism of *A. macrostemon* saponin.

In order to examine the way by which *A. macrostemon* saponin inhibits sCD40L-induced platelet activation, the present study identified several classical CD40-associated pathways, such as the PI3K/Akt, the MAPK and the NF-κB pathways. Initially, the present study verified the direct effects of sCD40L on the CD40-associated pathway. Consistent with our previous report regarding the investigation of the CD40L/CD40 signaling pathway, the involvement of additional proteins that interacted with the CD40-associated signaling pathway was confirmed, as expected. CD40 protein expression was upregulated in platelets treated with CD40L. This effect was accompanied with increased expression levels of both the PI3K/Akt and MAPK pathway proteins, such as PI3K, p-Akt, p-p38 and p-JNK. In addition, the expression levels of p-NF-κB were increased whereas those of IκB were decreased. These results verified that platelet activation was induced by CD40L. Subsequently, platelet pretreatment with saponin resulted in the opposite results of those noted following platelet treatment with CD40L. The costimulatory receptor/ligand complex CD40-CD40L plays an important role in atherosclerosis by inducing platelet activation and aggregation. The present study suggested the inhibition effect of *A. macrostemon* saponin on the CD40-related signaling pathway, which reduced platelet activation and inflammation leading to an anti-atherosclerotic effect.

In our previous study, we have determined that saponin inhibited the CD40-associated signaling pathway (Ou et al., 2012). However, the exact way by which saponin regulated these pathways was not clarified. The conjugation of TRAF proteins with CD40 plays an important role in the ubiquitination of platelets. CD40-TRAF2 interactions are crucial in platelets. The present study confirmed that saponin could reduce the expression levels of TRAF2, which promoted TRAF2 protein degradation. In contrast to these findings, saponin further downregulated the protein expression levels of ubiquitination proteins and specifically, those of k48 and k63. The present study confirmed that saponin exhibited significant effects on the inhibition of ubiquitination in platelets. For these reasons, we inferred that saponin regulated the CD40-associated signaling pathway by affecting the ubiquitination degradation induced by TRAF2. In order to further verify that the inhibitory effects of saponin on platelet activation are mediated by degradation of ubiquitination we established an MG132 platelet model to assess whether the effects of saponin on both protein degradation and on the release of the platelet activity marker TXB2 could be revered. The results confirmed that saponin regulated the CD40-related signaling pathway by affecting ubiquitination degradation, which in turn inhibited platelet activation, contributing to anti-inflammatory and antithrombotic effects during atherosclerosis development.

## Data Availability Statement

The raw data supporting the conclusions of this article will be made available by the authors, without undue reservation, to any qualified researcher.

## Ethics Statement

The use and care of patient samples were approved by the Ethics Committee of the Second Affiliated Hospital of Guangzhou Medical University. The patients/participants provided their written informed consent to participate in this study.

## Author Contributions

NL, WO, and SML designed the experiments. SSL, LJ, SZL, FZ, QX, ML, XC, XL, and JG performed the experiments, NL, WO wrote the manuscript. All authors read and approved the final manuscript.

## Funding

The study was supported by the Natural Science Foundation of Guangdong (1614050002543, 2020A1515010040), the Science and Technology Program of Guangzhou (202002030344), Guangzhou health and family planning science and technology project (20201A011082), Guangzhou high level clinical key specialty-cardiology (010R02113).

## Conflict of Interest

The authors declare that the research was conducted in the absence of any commercial or financial relationships that could be construed as a potential conflict of interest.
