# Supplementary material for: Allium macrostemon Saponin Inhibits Activation of Platelet via the CD40 Signaling Pathway
Source: Front Pharmacol. 2021 Jan 13;11:570603. doi: 10.3389/fphar.2020.570603 (PMC7874237; doi:10.3389/fphar.2020.570603)
Supplement: Supplementary file 1 [file datasheet1.docx]

**Chemicals**

The structures of Allium macrostemon saponin, new furostanol saponins(FS-1, FS-2 and FS-3), were reported previously as FS-1: (25R)-26-O-b- D -glucopyranosyl-5a-furostane-3b,12b, 22,26-tetraol-3-O-b- D -glucopyranosyl (1/2) [b- D -glucopyranosyl (1/3)]-b- D -glucopyranosyl (1/4)-b- D -galactopyranoside; FS-2: (25R)-26-O-b- D glucopyrano-syl-5a-furostane-3b,12a,22,26-tetraol-3-O-b- D -glucopyranosyl (1/2) [b-D -glucopyranosyl (1/3)]-b- D -glucopyranosyl (1/4)-b- D -galactopyranoside and FS-3: (25R)-26-O-b- D -glucopyranosyl-5b-furostane-3b,12a,22,26-tetraol-3-O-b- D -glucopyranosyl (1 / 2)-b- D -galactopyranoside(Ou et al., 2012).

**REFERENCES**

Ou, W.C., Chen, H.F., Zhong, Y., Liu, B.R., Liu, S.M., and Chen, K.J. (2012). Inhibition of platelet activation and aggregation by furostanol saponins isolated from the bulbs of Allium macrostemon Bunge. *Am J Med Sci* 344(4)**,** 261-267. doi: 10.1097/MAJ.0b013e31823ea9f0.


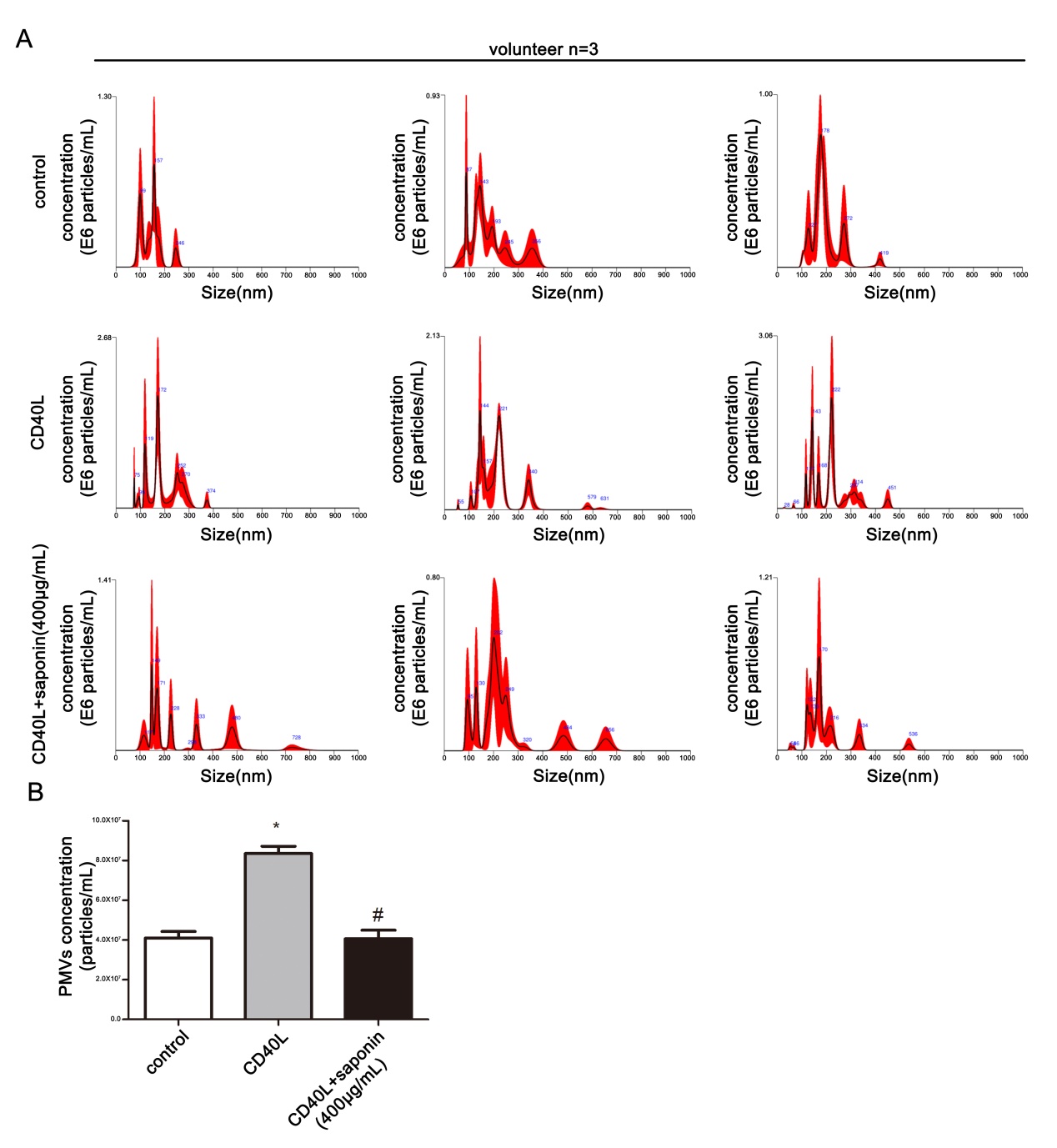


**FIGURE S1.** **Saponins inhibit platelet-derived microvesicles(PMVs) release**. (A, B) NTA detected PMVs from the platelets release, which peaked at 100 to 300nm.The chart showed the release concentration of PMVs in different treatment groups, *P<0.05 vs .control. ^#^P<0.05 vs. CD40L.


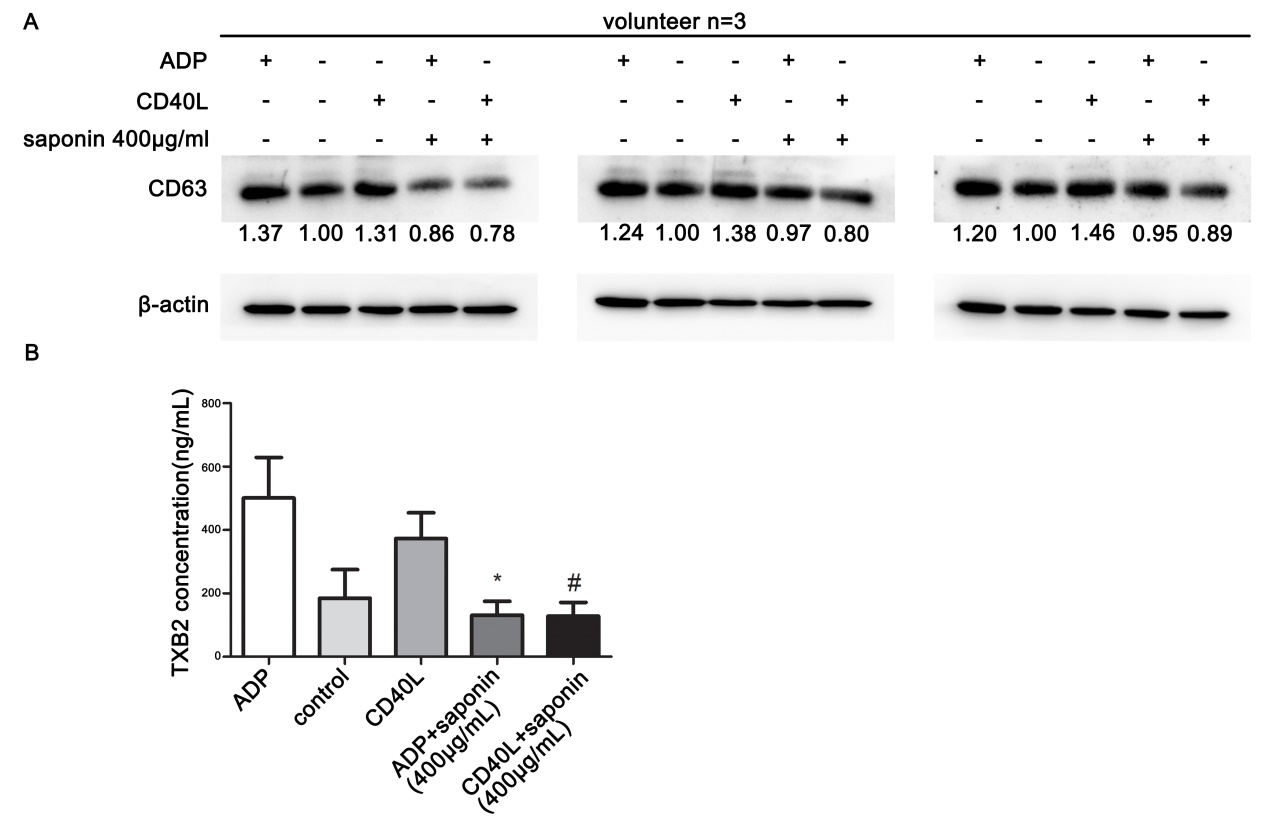


**FIGURE S2. Saponins inhibit platelet activation induced by CD40L.** (A) Western bloting method was used to detect the expression of CD63 protein in different treatment groups with β-actin as internal reference. (B) ELISA assay was used to detect the secretion of TXB2 in different treatment groups. *P<0.05 vs .ADP. ^#^P<0.05 vs. CD40L.
